# Reserve and Healthy Aging

**DOI:** 10.1093/geroni/igaa057.2878

**Published:** 2020-12-16

**Authors:** Cynthia Felix, Briana Sprague

## Abstract

In line with the GSA 2020 Annual Scientific Meeting theme of “Turning 75: Why Age Matters”, our symposium highlights the fact healthy aging is relevant to maintaining reserve- be it brain/cognitive reserve or physiological reserve. Even among older adults 75 or older, continuing to practice healthy aging habits, helps with reserve. In this symposium, Drs. Felix and Carlson discuss how positive neuroplastic processes such as social engagement and social volunteering may aid in brain/cognitive reserve. Dr. Lin discusses how negative neuroplastic processes such as hearing loss may hamper the same. The “use-it-or-lose-it” hypothesis may be a common pathway in effecting brain reserve, regardless of whether the inputs are social or sensory stimuli. Physiological reserve is also important in aging, and Dr. Sprague talks about energy and frailty, with frailty being an accelerated decline of physiological reserve. While the studies presented are from older adult populations, reserve often takes a lifetime of effort to build and maintain. The symposium speakers present several hypotheses such as brain reserve, cognitive reserve, cognitive load, information degradation, sensory deprivation and frailty. An application of these concepts, would help older adults practice aging habits that promote reserve, into advanced old age, at individual and community levels. Brain Interest Group Sponsored Symposium

